# XIAOPI Formula Inhibits Breast Cancer Stem Cells *via* Suppressing Tumor-Associated Macrophages/C-X-C Motif Chemokine Ligand 1 Pathway

**DOI:** 10.3389/fphar.2019.01371

**Published:** 2019-11-15

**Authors:** Shengqi Wang, Xiaoyan Liu, Renlun Huang, Yifeng Zheng, Neng Wang, Bowen Yang, Honglin Situ, Yi Lin, Zhiyu Wang

**Affiliations:** ^1^Integrative Research Laboratory of Breast Cancer, The Research Center for Integrative Medicine, Discipline of Integrated Chinese and Western Medicine & The Second Clinical College of Guangzhou University of Chinese Medicine, Guangzhou, China; ^2^Guangdong Provincial Key Laboratory of Clinical Research on Traditional Chinese Medicine Syndrome, Guangdong Provincial Academy of Chinese Medical Sciences, Guangdong Provincial Hospital of Chinese Medicine, Guangzhou, China; ^3^Post-doctoral Research Center, Guangzhou University of Chinese Medicine, Guangzhou, China; ^4^College of Basic Medicine, Guangzhou University of Chinese Medicine, Guangzhou, China

**Keywords:** XIAOPI formula, tumor-associated macrophages, breast cancer stem cells, C-X-C motif chemokine ligand 1, M2 phenotype polarization

## Abstract

Macrophages are the most abundant stromal cells associated with the host immune system in multiple malignancies including breast cancer. With proven clinical efficacy and no noticeable adverse effects, XIAOPI formula (XPS) has been approved for breast hyperplasia treatment by the State Food and Drug Administration of China (SFDA) in 2018. The existing knowledge about the anti-breast cancer activities and mechanisms of XPS has been very limited. The present study aimed to investigate whether XPS could exert an anti-breast cancer effect by regulating tumor-associated macrophages (TAMs) in tumor microenvironment. Herein, breast cancer cells and TAMs were co-cultured using the transwell co-culture system to simulate the coexistence of them. XPS could significantly inhibit the proliferation, colony formation, breast cancer stem cells (CSCs) subpopulation, mammosphere formation abilities as well as stemness-related genes expression in both human and mouse breast cancer cells in the co-culture system. Additionally, XPS could suppress M2 phenotype polarization as well as C-X-C motif chemokine ligand 1 (CXCL1) expression and secretion of TAMs. Notably, further mechanistic explorations verified TAMs/CXCL1 as the critical target of XPS in inhibiting breast CSCs self-renewal in the co-culture system as the exogenous CXCL1 administration could abrogate the inhibitory effect of XPS on breast CSCs self-renewal. More importantly, XPS significantly inhibited mammary tumor growth, breast CSCs subpopulation, and TAMs/CXCL1 activity in mouse 4T1-Luc xenografts *in vivo* without any detectable side effects. Taken together, this study not only uncovers the immunomodulatory mechanism of XPS in treating breast cancer but also sheds novel insights into TAMs/CXCL1 as a potential molecular target for breast CSCs elimination.

## Introduction

Breast cancer is the most commonly diagnosed malignancy and the second leading cause of cancer-related deaths among women (Allemani et al., 2015; Bray et al., 2018). Breast cancer alone accounts for approximately 24.2% of new cancer diagnoses and 15.0% of cancer-related deaths in women worldwide (Bray et al., 2018). The incidence of breast cancer is still increasing (Allemani et al., 2015) and the onset age of breast cancer is tending younger globally (Ali et al., 2011). Although the overall survival of breast cancer patients has been significantly improved over the past few decades due to early detection and multidisciplinary progress including conventional surgery, radiotherapy, chemotherapy, endocrine therapy, and molecular targeted therapy, a considerable proportion of patients will still suffer from relapse or even die of breast cancer (Chia et al., 2008). Thus, extensive efforts are being made to identify novel treatment targets or to develop novel drugs for this terrible disease worldwide.

With encouraging efficacy in prolonging survival time, alleviating side effects, and improving the quality of life in cancer patients, Traditional Chinese Medicine (TCM) has been widely used for the prevention and treatment of cancer for thousands of years, mainly in China and surrounding areas (Nie et al., 2016; Liao et al., 2017). Notably, TCM has unique advantages in breast cancer treatment and could improve short-term treatment efficacy, extend 5-year survival, and reduce the incidence of adverse reactions in breast cancer patients after mastectomy (Tian et al., 2015; Wang et al., 2015). TCM has been widely used for breast cancer treatment in the clinic. It was estimated that more than 35.6% of breast cancer patients in Taiwan used to receive TCM treatment (Lin and Chiu, 2011). XIAOPI formula (XPS) is a new anti-mammary hyperplasia drug approved by the SFDA in Dec 28, 2018. It has been empirically used for mammary hyperplasia treatment in the clinic for several decades and its therapeutic efficacy has been confirmed by extensive clinical practices. Breast cancer usually originates from the uncontrolled proliferation of mammary epithelial cells and mammary-ductal hyperplasia, while atypical hyperplasia is a premalignant lesion of the breast (Santisteban et al., 2010). Additionally, network-pharmacology analysis indicated that 10 herbs in XPS might contain 105 active compounds and further modulate 806 potential targets, 81 of which were closely correlated with breast cancer development (Wang et al., 2017). Based on the above evidence, we speculate that XPS may also be efficient in inhibiting breast cancer. However, the existing knowledge about the anti-breast cancer activities and mechanisms of XPS has been very limited.

In TCM philosophy, breast cancer is regarded as a systemic disease which results from the imbalance of cellular microenvironment. Therefore, TCM focuses on the holistic enhancement of inner immunity and remodeling tumor immune microenvironment for breast cancer treatment, but not directly killing cancer cells (Cohen et al., 2002; Xie et al., 2018). Breast CSCs are a subgroup of tumor cells that are characterized by their tumor-initiating capacity, self-renewal capacity, pluripotency, and high tumorigenicity (Vahidian et al., 2019). Increasing evidence has implicated that breast CSCs is essential for breast cancer development, progression, recurrence, and treatment resistance (Shima et al., 2017). Therefore, more targeted strategies on the scope of breast CSCs are in urgent need to improve the prognosis of breast cancer patients. Accumulating evidence has indicated that CSCs can escape immune destruction and foster establishment of the immunosuppressive tumor microenvironment by recruiting and interacting with a broad range of immune cells (Vahidian et al., 2019). Therefore, further elucidation of breast CSCs-immune cell interactions and the underlying signaling mechanisms will open up novel opportunities to improve breast cancer immunotherapy (Vahidian et al., 2019). The tumor immune microenvironment consists of multiple immune cells, including macrophages, natural killer cells, myeloid-derived suppressor cells (MDSCs), regulatory T cells (Tregs), cytotoxic T-lymphocytes (CTLs), and T helper (Th) cells (Binnewies et al., 2018). Macrophages are the most abundant immune cells in multiple malignancies including breast cancer (Lao et al., 2017; De la Fuente Lopez et al., 2018). Macrophages are generally categorized into two broads but distinct subsets as either classically activated (M1) or alternatively activated [(M2 or Tumor-associated macrophages (TAMs)] (Parisi et al., 2018). M2 type macrophages predominate in human cancers and produce growth-promoting chemokines that actively stimulate tumor growth. TAMs infiltration creates an inflammatory condition that supports cancer initiation and secretes multiple cytokines to promote cancer angiogenesis and invasion and suppresses antitumor immunity (Mills et al., 2016). TAMs have also been implicated in the promotion of breast cancer growth and metastasis (Hollmen et al., 2016). Clinical evidence has compellingly demonstrated the association between high TAMs infiltration and poor prognosis in patients with breast cancer (Tang, 2013; Hollmen et al., 2016). Nowadays TCM is being paid much attention because of its therapeutic significance in cancer treatment. Inhibiting TAMs toward M2 type polarization or reprogramming TAMs to M1 type have been reported to inhibit cancer growth by multiple studies (Parisi et al., 2018). However, it is still unclear whether XPS could inhibit macrophages M2 polarization or their cytokines secretion in the tumor immune microenvironment.

In the present study, by establishing the TAMs-breast cancer cells co-culture system *in vitro* and breast cancer xenografts *in vivo*, we systematically demonstrated that XPS could suppress M2 phenotype polarization as well as CXCL1 expression and secretion, which eventually led to the inhibitory effect of XPS on the self-renewal of breast CSCs. Overall, our study uncovers the immunomodulatory mechanism of XPS in treating breast cancer and highlights TAMs/CXCL1 as a potential molecular target for breast CSCs elimination.

## Materials and Methods

### Cell Culture and Induction

The non-malignant human mammary epithelial cell line MCF-10A, human breast cancer cell line MDA-MB-231, mouse breast cancer cell line 4T1, human acute monocytic leukemia cell line Thp1 and mouse macrophage cell line Raw264.7 were obtained from the American Type Culture Collection. The identities of all these cell lines have been authenticated by short tandem repeat profiling. MCF-10A cells were cultured in DMEM/F12 medium supplemented with 5% horse serum, 1% penicillin and streptomycin (Gibco, Grand Island, NY, USA), 20 ng/ml recombinant human epidermal growth factor (EGF), 0.5 µg/ml hydrocortisone, 100 ng/ml cholera toxin, and 10 µg/ml insulin (Sigma-Aldrich, Shanghai, China). MDA-MB-231 and 4T1 cells were cultured in DMEM medium supplemented with 10% fetal bovine serum and 1% penicillin and streptomycin (Gibco). Thp1 and Raw264.7 cells were cultured in RPMI-1640 medium supplemented with 10% fetal bovine serum and 1% penicillin and streptomycin (Gibco). All the cells were maintained at 37°C in a humidified incubator containing 5% CO_2_. 100 ng/ml phorbol-12-myristate-13-acetate (PMA, Sigma-Aldrich) was used to induce attachment and differentiation of Thp1 monocytes into macrophages. The cell culture supernatant of breast cancer cell lines MDA-M-231 and 4T1 was collected as conditioned medium (CM), and was used to induce the transformation of Thp1 macrophages and RAW264.7 macrophages into M2 phenotype TAMs, respectively. For co-culture of breast cancer cells and TAMs, the 6-well or 24-well transwell co-culture system was used. In brief, transwell inserts were placed in 6-well or 24-well culture plates seeded with breast cancer cells. TAMs were placed in the upper transwell chamber. Transwell inserts were separated by a 0.4 µm permeable membrane allowed free exchange of media and soluble molecules.

### Preparation and Quality Control of XPS

XPS was extracted from a mixture of 10 herbs including *Epimedium brevicornum* Maxim. (Chinese name Yin Yang Huo), *Cistanche deserticola* Ma (Chinese name Rou Cong Rong), *Leonurus japonicus* Houtt. (Chinese name Yi Mu Cao), *Salvia miltiorrhiza* Bunge (Chinese name Dan Shen), *Curcuma aromatica* Salisb. (Chinese name Yu Jin), *Curcuma longa* L. (Chinese name E Zhu), *Ligustrum lucidum* W.T.Aiton (Chinese name Nv Zhen Zi), *Reynoutria multiflora* (Thunb.) Moldenke (Chinese name He Shou Wu), *Crassostrea gigas* (Chinese name Mu Li) and *Carapax trionycis* (Chinese name Bie Jia) by refluxing extraction method. Its quality control was carried out by detecting the high performance liquid chromatography fingerprints between different batches. The detailed preparation and quality control information have been previously reported (Liu, 2010; Wang and Huang, 2010; Wang et al., 2017).

### Flow Cytometry Assay

The phenotype of macrophages was identified by analyzing the surface markers of macrophages using flow cytometer. Briefly, macrophages were harvested, washed, and resuspended in 100 µl PBS solution at a density of 5 × 10^6^ cells/ml. For Thp1 macrophages, cells were incubated with FITC-conjugated F4/80 antibody (SC-71085, Santa Cruz Biotechnology, Santa Cruz, CA, USA) and PE-conjugated CD163 antibody (12-1639-42, eBioscience, San Diego, CA, USA) for 30 mins at 37°C. For Raw264.7 macrophages, cells were incubated with FITC-conjugated F4/80 antibody (SC-71085, Santa Cruz) and PE-conjugated CD206 antibody (141705, Biolegend, San Diego, CA, USA) for 30 mins at 37°C. For phenotype analysis of primary macrophages extracted from mouse 4T1-Luc xenografts, cells were incubated with CD45-PE-Cy7 (25-0451-82, eBioscience), FITC-conjugated F4/80 antibody (SC-71085, Santa Cruz) and PE-conjugated CD206 antibody (141705, Biolegend) for 30 mins at 37°C. After incubation, cells were washed once with PBS and subjected to analysis using a FC500 flow cytometry (Beckman Coulter, Fullerton, CA, USA) or a FACSAria III flow cytometer (BD Biosciences, San Diego, CA, USA). The F4/80^+^/CD163^+^ subpopulation, the F4/80^+^/CD206^+^ subpopulation or the CD45^+^/F4/80^+^/CD206^+^ subpopulation were quantified and defined as M2 phenotype macrophages.

### MTT Assay

MTT assay was applied to assess the cytotoxicity of XPS in mammary epithelial cells, breast cancer cells and macrophages. In brief, cells were seeded into 96-well plates at a density of 5×10^3^ cells/well. After cells attachment, cells were treated with serial concentration gradients of XPS for 24 h or 48 h. To investigate whether XPS still has an inhibitory effect on proliferation of breast cancer cells in the presence of TAMs, breast cancer cells and TAMs were co-cultured in the 24-well transwell co-culture system. TAMs were seeded in the upper transwell chamber at a density of 3 × 10^4^ cells per well while breast cancer cells were seeded in the lower chamber at a density of 5 × 10^3^ cells per well. After cells attachment, cells were treated with serial concentration gradients of XPS for 48 h. Cell viability was detected using MTT (MP Biomedicals, Shanghai, China) according to the manufacturer’s instructions. MTT assay was performed in independent triplicates.

### Colony Formation Assay

To investigate the effect of XPS on colony formation of breast cancer cells, MDA-MB-231, and 4T1 cells were seeded in 6-well plate at a density of 2,000 cells per well. To investigate whether XPS still has an inhibitory effect on colony formation of breast cancer cells in the presence of TAMs, breast cancer cells and TAMs were co-cultured in the 24-well transwell co-culture system. TAMs were seeded in the upper transwell chamber at a density of 3s × 10^4^ cells per well while breast cancer cells were seeded in the lower chamber at a density of 200 cells per well. After attachment, cells were treated with serial concentration gradients of XPS for 48 h and then replaced with fresh medium. Then, cells were cultured for 2 weeks and the resultant colonies were fixed with 4% paraformaldehyde and then stained with coomassie blue solution.

### Breast CSCs Population Analysis

Breast CSCs populations were analyzed by flow cytometry using the ALDEFLUOR Stem Cell Identification Kit (01700, STEMCELL Technologies, Vancouver, Canada) according to the manufacturer’s instructions. Briefly, breast cancer cells were seeded in a 6-well plate at a density of 4 × 10^5^ cells/well. For co-culture of breast cancer cells and TAMs, 4s × 10^5^ TAMs were seeded in the upper transwell chamber and the transwell inserts were placed in the 6-well culture plates seeded with 4×10^5^ breast cancer cells/well. Then, cells were treated with indicated concentrations of XPS, CXCL1 (Human CXCL1, C597, Novoprotein, Shanghai, China; Murine CXCL1, 250-11-20, Peprotech, Rocky Hill, NJ, USA) or XPS and CXCL1 combination for 48 h. After treatment, breast cancer cells were harvested and resuspended in 500 µl ALDEFLUOR™ assay buffer. Then, cells were incubated with ALDEFLUOR™ at 37°C for 30 min to mark ALDH^+^ cells. After incubation, cells were washed once with PBS and subjected to analysis using the FC500 flow cytometer (Beckman). The ALDH^+^ subpopulation was defined as breast CSCs. Diethylaminobenzaldehyde (DEAB), a specific inhibitor of ALDH activity, was used to control for background fluorescence in the ALDH staining assay. For breast CSCs population analysis in the mouse 4T1-Luc xenografts, primary breast cancer cells were firstly isolated from mouse mammary tumors by mechanical methods and then subjected to breast CSCs population analysis as indicated above.

### Mammosphere Formation Assay

Breast cancer cells were seeded in ultralow attachment 6-well plate at a density of 1 × 10^5^ cells/well. For co-culture of breast cancer cells and TAMs, 4 × 10^5^ TAMs were seeded in the upper transwell chamber and the transwell inserts were placed in the 6-well culture plates seeded with 1 × 10^5^ breast cancer cells/well. Then, the above cells were treated with XPS, CXCL1 or XPS and CXCL1 combination for 48 h as indicated. The complete culture medium for mammosphere formation assay was composed of DMEM/F12 medium supplemented with 1% penicillin-streptomycin (Gibco), 2% B27 supplement (Gibco), 20 ng/ml EGF, 5 µg/ml insulin and 0.4% bovine serum albumin (BSA, Sigma-Aldrich). The numbers of mammospheres were quantified microscopically.

### QPCR

Total RNA was extracted with Trizol and reverse transcribed to complementary cDNA using the PrimeScript™ RT reagent Kit (Takara, Shiga, Japan) in accordance with the manufacturer’s instructions. RT-PCR was performed using the SYBR Premix Ex Taq Kit (Takara) and the QuantStudio 6&7 Flex Real-Time PCR System (Applied Biosystems, Foster City, CA, USA). Primer sequences of human *β-actin* were 5′-CCAACCGCGAGAAGATGA-3′ (forward) and 5′-CCAGAGGCGTACAGGGATAG-3′ (reverse). Primer sequences of human *β-catenin* were 5′-GCTTTCAGTTGAGCTGACCA-3′ (forward) and 5′-CAAGTCCAAGATCAGCAGTCTC-3′ (reverse). Primer sequences of human *OCT4* were 5′-CAATTTGCCAAGCTCCTGA-3′ (forward) and 5′-AGATGGTCGTTTGGCTGAAT-3′ (reverse). Primer sequences of human *Nanog* were 5′-ATGCCTCACACGGAGACTGT-3′ (forward) and 5′-CAGGGCTGTCCTGAATAAGC-3′ (reverse). Primer sequences of human *CXCL1* were 5′-AGGGAATTCACCCCAAGAAC-3′ (forward) and 5′-ACTATGGGGGATGCAGGATT-3′ (reverse). Primer sequences of mouse *β-actin* were 5′-GGAGGGGGTTGAGGTGTT-3′ (forward) and 5′-GTGTGCACTTTTATTGGTCTCAA-3′ (reverse). Primer sequences of mouse *β-catenin* were 5′-ACAGCACCTTCAGCACTCT-3′ (forward) and 5′-AAGTTCTTGGCTATTACGACA-3′ (reverse). Primer sequences of mouse *OCT4* were 5′-CTGTAGGGAGGGCTTCGGGCACTT-3′ (forward) and 5′-CTGAGGGCCAGGCAGGAGCACGAG-3′ (reverse). Primer sequences of mouse *Nanog* were 5′-AGGGTCTGCTACTGAGATGCTCTG-3′ (forward) and 5′-CAACCACTGGTTTTTCTGCCACCG-3′ (reverse). Primer sequences of mouse *CXCL1* were 5′-GACTCCAGCCACACTCCAAC-3′ (forward) and 5′-TGACAGCGCAGCTCATTG-3′ (reverse).

### Western Blotting

Cells were treated as indicated and then lysed using RIPA (Beyotime Biotechnology, Shanghai, China). Protein concentration was quantified with the Bicinchoninic Acid Kit (Sigma-Aldrich) according to the manufacturer’s instructions. Equal amounts of protein (30 µg) were resolved on SDS-PAGE gel, transferred to a polyvinylidene fluoride microporous membrane (Millipore, Billerica, MA, USA) and probed with primary antibodies for β-actin (4970S, Cell Signaling Technology, CST, Boston, MA, USA), CXCL1(AF5403, Affinity Biosciences, Cincinnati, OH, USA), and β-catenin (51067-2-AP, Proteintech, Chicago, USA). The relative expression levels of proteins between groups were calculated by comparing optical densities of bands using the Gel-Pro analyzer four software (Media Cybernetics, Maryland, USA).

### Elisa Assay

The effect of XPS on CXCL1 secretion from TAMs was detected by Elisa assay. Briefly, TAMs were cultured in a 6-well plate and treated with serial concentration gradients of XPS for 48 h. After XPS treatment, TAMs were harvested and seeded into 6-well plate again in a density of 5 × 10^5^ cells/well and cultured for 24 h. Then, the cell culture supernatant in each well was collected and CXCL1 concentration in the cell culture supernatant was detected using the Human CXCL1 Elisa Kit (SEA041Hu, USCN Business, Wuhan, China) or Mouse CXCL1 Elisa Kit (SEA041Mu, USCN Business) according to the manufacturer’s instructions.

### Double Luciferase Reporter Gene Assay

The effect of XPS on *CXCL1* promoter activity was investigated by double luciferase reporter gene assay. Briefly, the CXCL1 promoter plasmid (Genecopeia, Guangzhou, China) was transfected into Raw264.7-derived TAMs using Vigenefection (FH880806, Vigene Biosciences, Jinan, China) according to the manufacturer’s instructions. Then, the transfected cells were seeded into 96-well plate at a density of 2 × 10^4^ cells/well and treated with serial concentration gradients of XPS for 48 h. After XPS treatment, the cell culture supernatant in each well was collected. The CXCL1 promoter activity was detected by analyzing the Gaussia luciferase activity and secreted alkaline phosphatase activity in the cell culture supernatant using the Secrete-Pair™ Dual Luminescence Assay Kit (LF031, Genecopeia) according to the manufacturer’s instructions.

### Tissue Immunofluorescence

For immunofluorescence analysis, frozen tissue sections of breast tumor were fixed with 4% paraformaldehyde for 20 mins, washed three times with PBS, permeabilized with 0.25% Triton X-100 for 20 mins and then blocked in 5% BSA at room temperature for 1 h. For F4/80 and CXCL1 detection, tissue sections were incubated with F4/80 antibody (17-4801-80, eBioscience) and CXCL1 antibody (AF5403, Affinity) overnight at 4°C, followed by an incubation with the Alexa Fluor^®^ 555 conjugated-anti-rat IgG (4417S, CST) and the Alexa Fluor^®^ 488 conjugated-anti-rabbit IgG (4412S, CST) for 2 h. For ALDH1A1 detection, tissue sections were incubated with ALDH1A1 antibody (BF0220, Affinity) overnight at 4°C, followed by an incubation with the secondary antibody of Alexa Fluor^®^ 555 conjugated-anti-mouse IgG (A21422, ThermoFisher) for 2 h. 4’, 6-diamidino-2-phenylindole (DAPI, Sigma) was used to visualize the nuclei. Fluorescence images were obtained using the LSM710 confocal microscope (Zeiss, Jena, Germany).

### Animal Experiment

All *in vivo* experiments were performed according to our institution’s guidelines for the use of laboratory animals and were approved by the Institutional Animal Care and Use Committee of Guangdong Provincial Hospital of Chinese Medicine (No.2018044). Six-week-old female Balb/c mice were raised at the Experimental Animal Center of Guangdong Provincial Hospital of Chinese medicine under specific pathogen-free conditions with ambient temperature of 20-25°C and 45%–50% relative humidity and given sterilized food and water. The rearing facility was maintained on a 12 h light-dark cycle. For 4T1-Luc xenografts establishment, 2×10^6^ 4T1-Luc cells were suspended in 200 µl PBS and inoculated subcutaneously into the mammary fat pads of mice. When tumors reached a mean diameter of 0.5 cm, mice were randomized into two groups (six in each group) and received either saline or XPS (1 g/kg/day) by intragastric perfusion. Throughout the treatment, mice were weighed and their tumors were measured with a caliper every 3 days. Tumor volumes (V) were calculated using the formula: V = (length) × (width)^2^/2. D-Luciferin (150 mg/kg weight, 122799, PerkinElmer, Boston, USA) was injected intraperitoneally and mice were imaged using the IVIS Lumina XR *in vivo* imaging system (PerkinElmer) every week to monitor tumor growth and metastasis. When tumors grew to a proper size, mice were euthanized and tumors were excised. Primary cells were isolated from fresh mammary tumors by mechanical methods and subjected for macrophages phenotype analysis or breast CSCs subpopulation analysis as indicated above. The remaining tumor tissue was stored at -80°C and used for tissue immunofluorescence assay as indicated above.

### Statistical Analysis

Data was presented as mean ± standard deviation (SD). All statistical analyses were performed using SPSS 19.0 software (Abbott Laboratories, Chicago, USA). Student’s *t*-test and one-way ANOVA were performed for comparisons among groups. *P* < 0.05 was considered to be statistically significant.

## Results

### XPS Significantly Inhibits the Proliferation and Colony Formation Abilities of Breast Cancer Cells in the Co-Culture System

**Figure 1:**
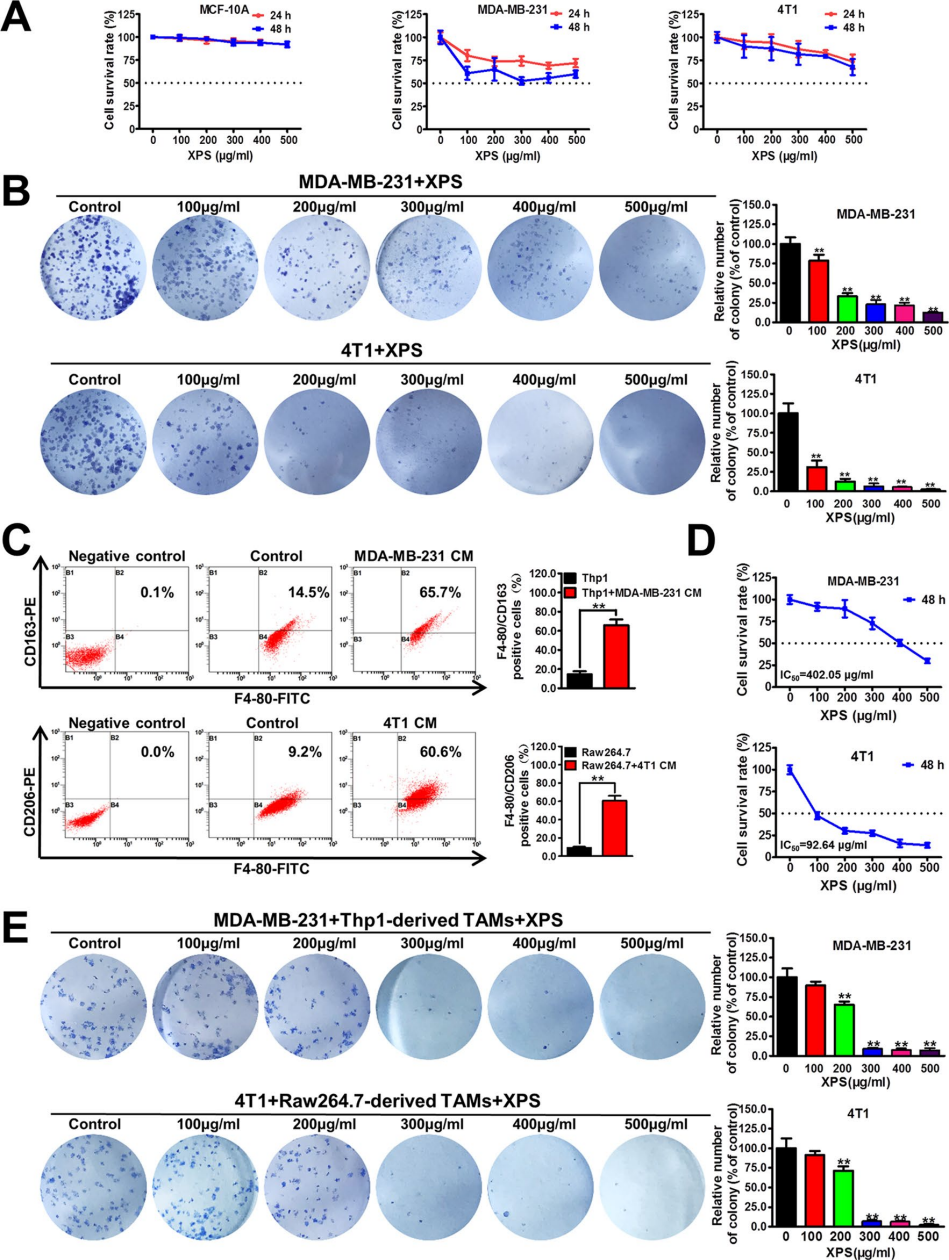
XIAOPI formula (XPS) significantly inhibits the proliferation and colony formation abilities of breast cancer cells in the co-culture system. **(A)** The cytotoxicity of XPS in nonmalignant mammary epithelial cell line MCF-10A and breast cancer cell lines MDA-MB-231 and 4T1 was investigated by MTT method. **(B)** XPS (100∼500 μg/ml) could dramatically inhibit the colony formation abilities of breast cancer cell lines MDA-MB-231 and 4T1. **(C)** Both human and mouse macrophages were successfully induced into M2 phenotype TAMs when they were treated with the conditional medium (CM) of breast cancer cells for 48 h. **(D)** XPS treatment for 48 h could dramatically inhibit the proliferation of breast cancer cell lines MDA-MB-231 and 4T1 in the co-culture system. The IC_50_ values were calculated by Bliss method. **(E)** XPS could significantly suppress the colony formation abilities of breast cancer cell lines MDA-MB-231 and 4T1 in the co-culture system. All values are presented as the mean ± SD, n = 3, **P < 0.05.

Firstly, the cytotoxicity of XPS in both nonmalignant mammary epithelial cells and breast cancer cells was investigated by MTT method. As shown in **Figure 1A**, XPS treatment for 24 h or 48 h could only moderately inhibit the proliferation of human breast cancer cell line MDA-MB-231 and mouse breast cancer cell line 4T1, while having no obvious cytotoxicity in MCF-10A cells. The IC_50_ values of XPS in these three kinds of cells reached over 500 µg/ml. However, XPS could dramatically inhibit the colony formation abilities of breast cancer cell lines MDA-MB-231 and 4T1 during the 2-week culture period (**Figure 1B**), indicating a long-term inhibitory effect of XPS on breast cancer cell proliferation. In the tumor microenvironment, cancer cells coexist with multiple kinds of cells, of which the largest proportion is TAMs. TAMs often display an immune-suppressive M2-like phenotype that fosters tumor growth and promotes resistance to therapy. In the present study, the conditional medium (CM) of MDA-MB-231 cells significantly increased the F4/80^+^/CD163^+^ subpopulation in Thp1 macrophages while 4T1-CM significantly elevated the F4/80^+^/CD206^+^ population in mouse Raw264.7 macrophages. These results indicated that both human Thp1 macrophages and mouse Raw264.7 macrophages were successfully stimulated into M2 phenotype macrophages (**Figure 1C**). Breast cancer cells were co-cultured with these M2-like phenotype TAMs using the transwell co-culture system to investigate whether XPS could inhibit the growth of breast cancer cells in the presence of TAMs. As shown in **Figure 1D**, XPS treatment for 48 h could dramatically inhibit the proliferation of breast cancer cell lines MDA-MB-231 and 4T1 in the co-culture system.The IC_50_ values of XPS were 402.05 µg/ml for MDA-MB-231 cells and 92.64 µg/ml for 4T1 cells respectively when they were co-cultured with TAMs. Meanwhile, XPS could also strongly suppress the colony formation abilities of breast cancer cell lines MDA-MB-231 and 4T1 in the co-culture system (**Figure 1E**). Altogether, XPS could significantly inhibit the proliferation and colony formation abilities of breast cancer cells particularly when they were co-cultured with TAMs.

### XPS Inhibits the Self-Renewal Activity of Breast CSCs in the Breast Cancer Cells and TAMs Co-Culture System

**Figure 2:**
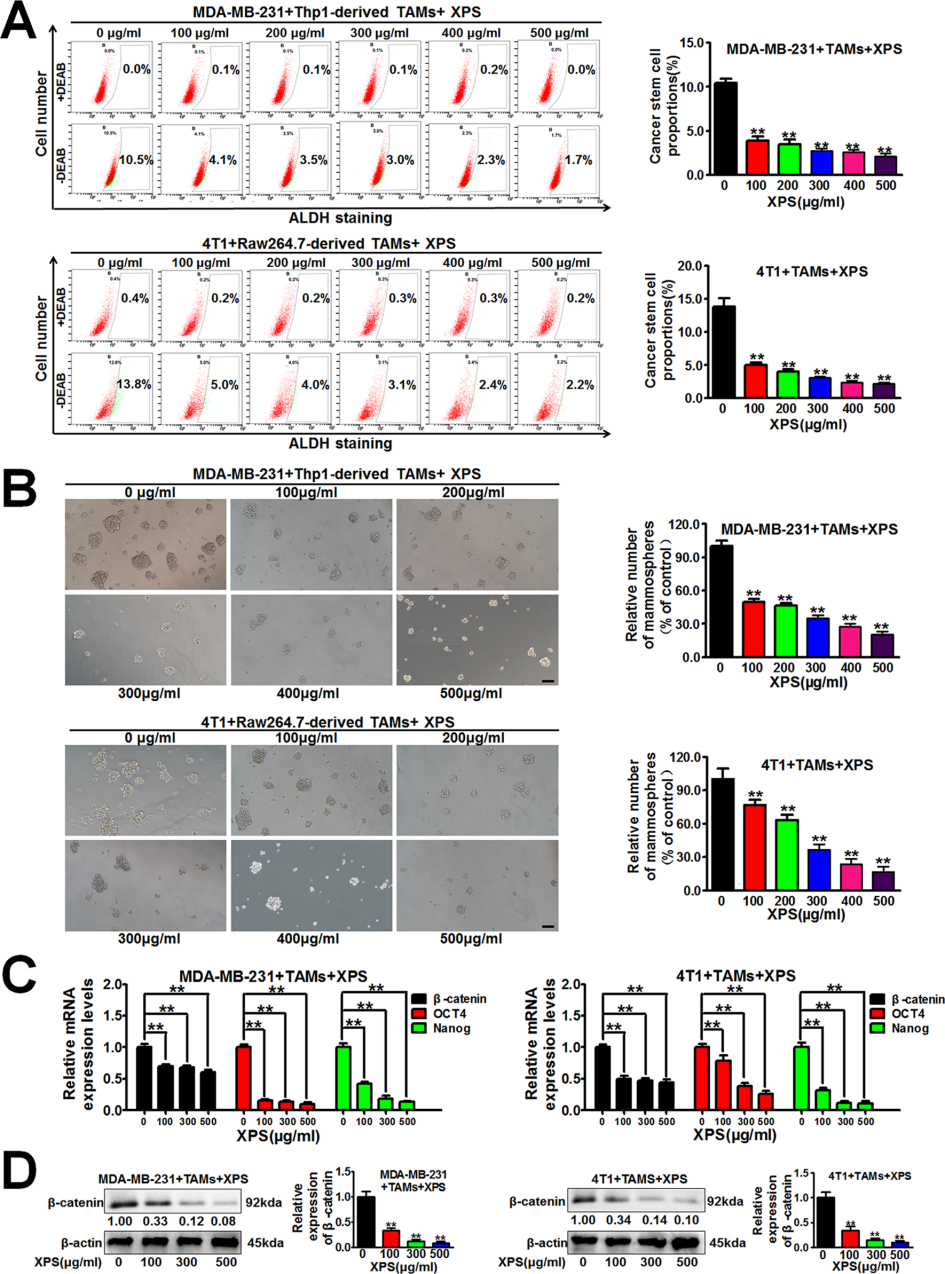
XIAOPI formula (XPS) inhibits the self-renewal activity of breast cancer stem cells (CSCs) in the breast cancer cells and tumor-associated macrophages (TAMs) co-culture system. **(A)** XPS treatment for 48 h could significantly decrease the proportions of ALDH^+^ subpopulations in both the MDA-MB-231 cells and 4T1 cells when they were co-cultured with TAMs. **(B)** XPS treatment for 48 h significantly decreased the mammospheres’ numbers of the co-cultured breast cancer cells. Scale bar = 100 μm. **(C)** XPS treatment for 48 h strongly attenuated the mRNA expression levels of *β-catenin*, *OCT4*, and *Nanog* in the co-cultured breast cancer cells. **(D)** XPS treatment for 48 h could dramatically attenuate β-catenin protein expression levels in the co-cultured breast cancer cells. All values are presented as the mean ± SD, n = 3, **P < 0.05.

Breast CSCs are considered to be the initiator cell which is responsible for breast cancer cell proliferation and growth. Therefore, we further investigated the influence of XPS on breast CSCs. Hyperactive aldehyde dehydrogenase (ALDH) activity is closely related to the physiological properties of CSCs (Wang and Lei, 2017) and the ALDH^+^ subpopulation exhibits enhanced stemness when compared with the ALDH^-^ subpopulation (Shao et al., 2016). As shown in **Figure 2A**, XPS could significantly decrease the proportions of ALDH^+^ subpopulations in both MDA-MB-231 cells and 4T1 cells when they were co-cultured with TAMs. Breast CSCs are enriched in non-adherent spherical clusters of cells, which are termed mammospheres (Wang et al., 2014). Therefore, we further investigated if XPS could suppress the mammosphere formation efficacy of breast CSCs in the breast cancer cells and TAMs co-culture system. As shown in **Figure 2B**, XPS treatment significantly decreased the mammosphere number in the breast cancer cells and TAMs co-culture system. It has been reported that multiple stem cell transcription factors are overexpressed in CSCs, such as octamer-binding transcription factor 4 (*OCT4*), *Nanog*, and *β-catenin*, which maintain pluripotency (Hattermann et al., 2016). To further verify the inhibitory effect of XPS on breast CSCs in the co-culture system, QPCR assay was applied to investigate the effect of XPS on the expression levels of stemness-related genes in these co-cultured breast cancer cells. As shown in **Figure 2C**, XPS strongly attenuated the expression levels of stemness-related genes, including *β-catenin*, *OCT4*, and *Nanog*, in the co-cultured breast cancer cells. As well known, β-catenin signaling is associated with increased stemness activity and chemoresistance of breast CSCs. In addition, it has been widely reported that β-catenin could directly upregulate *OCT4* (Kelly et al., 2011; Lee et al., 2014; Qi et al., 2016; Yong et al., 2016) and *Nanog* (Takao et al., 2007; Li et al., 2013; Yong et al., 2016; Yu et al., 2017) gene transcription and protein expression in multiple cancer cells and CSCs. Therefore, we only further investigated whether XPS could attenuate the protein expression level of β-catenin. As shown in **Figure 2D**, western blot assay further convinced that XPS could also dramatically attenuate β-catenin protein expression levels in the co-cultured breast cancer cells. Altogether, XPS could inhibit the self-renewal activity of breast CSCs in the breast cancer cells and TAMs co-culture system.

### XPS Inhibits M2 Phenotype Polarization, CXCL1 Expression and Secretion of TAMs

**Figure 3:**
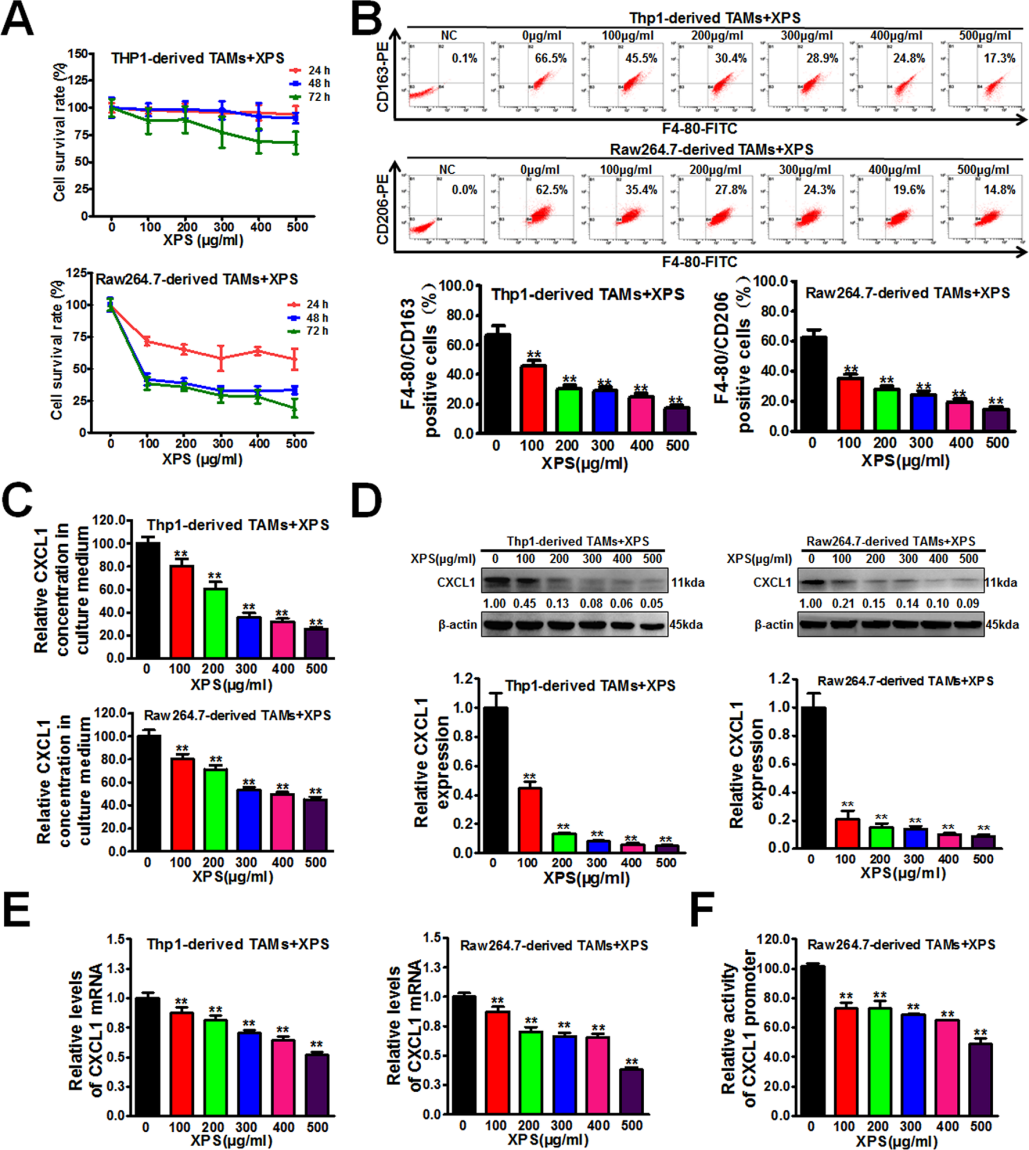
XIAOPI formula (XPS) inhibits M2 phenotype polarization, C-X-C motif chemokine ligand 1 (CXCL1) expression and secretion of tumor-associated macrophages (TAMs). **(A)** MTT assay indicated that XPS (100∼500 μg/ml) could strongly inhibit the proliferation of Raw264.7-derived TAMs while exhibiting no obvious cytotoxicity in Thp1-derived TAMs. **(B)** XPS treatment for 48 h could dramatically inhibit M2 phenotype polarization of TAMs induced by CM of breast cancer cells. **(C)** Elisa assay indicated that XPS treatment for 48 h could significantly inhibit CXCL1 secretion from TAMs in a dose-dependent manner. **(D**–**E)** Western blot and QPCR results further verified that XIAOPI formula treatment for 48 h could dramatically inhibit CXCL1 protein expression levels **(D)** and *CXCL1* mRNA transcription levels **(E)** in both human and mouse TAMs. **(F)** Double luciferase reporter gene assay suggested that XPS treatment for 48 h could suppress the promoter activity of *CXCL1* gene in Raw264.7-derived TAMs. All values are presented as the mean ± SD, n = 3, **P < 0.05.

Extensive researches have reported that TAMs in the tumor microenvironment could foster tumor growth. Therefore, we speculated that XPS could inhibit breast cancer growth by modulating TAMs. As shown in **Figure 3A**, XPS could dramatically inhibit the proliferation of Raw264.7-derived TAMs while exhibiting no obvious cytotoxicity in Thp1-derived TAMs. These results indicated that XPS might regulate TAMs through other mechanisms rather than direct cytotoxicity effects. TAMs often display a pro-tumorigenic M2 phenotype that fosters tumor growth and promotes resistance to therapy. Yet, macrophages are highly plastic and can also transform into anti-tumorigenic M1 phenotype (Parisi et al., 2018). As shown in **Figure 3B**, XPS could dramatically inhibit M2 phenotype polarization of TAMs induced by CM of breast cancer cells. Next, it is important to investigate how XPS-induced TAMs polarization variations brought into robust changes in breast CSCs population in the co-culture system. Since breast cancer cells and TAMs were separated by 0.4 µm permeable membrane in the transwell co-culture system, which only allowed free exchange of media and soluble molecules, but forbade direct cell interaction, we therefore speculated that some pro-tumorigenic molecules secreted by TAMs may be suppressed by XPS and subsequently weakened the promotion effect of TAMs on breast CSCs self-renewal. CXCL1 was the most-abundant chemokine secreted by TAMs (Palomino and Marti, 2015; Wang et al., 2018). Elisa assay indicated that XPS could significantly inhibit CXCL1 secretion from TAMs in a dose-dependent manner (**Figure 3C**). Western blot and QPCR results further verified that XIAOPI formula could dramatically inhibit CXCL1 protein expression levels (**Figures 3D, E**) and mRNA transcription in a dose-dependent manner in both human and mouse TAMs (**Figure 3F**). Double luciferase reporter gene assay suggested that XPS suppressed the promoter activity of *CXCL1* gene in TAMs. Altogether, XPS could inhibit M2 phenotype polarization and CXCL1 expression and secretion of TAMs.

### XPS Suppresses Breast CSCs by Modulating TAMs/CXCL1 Pathway

**Figure 4:**
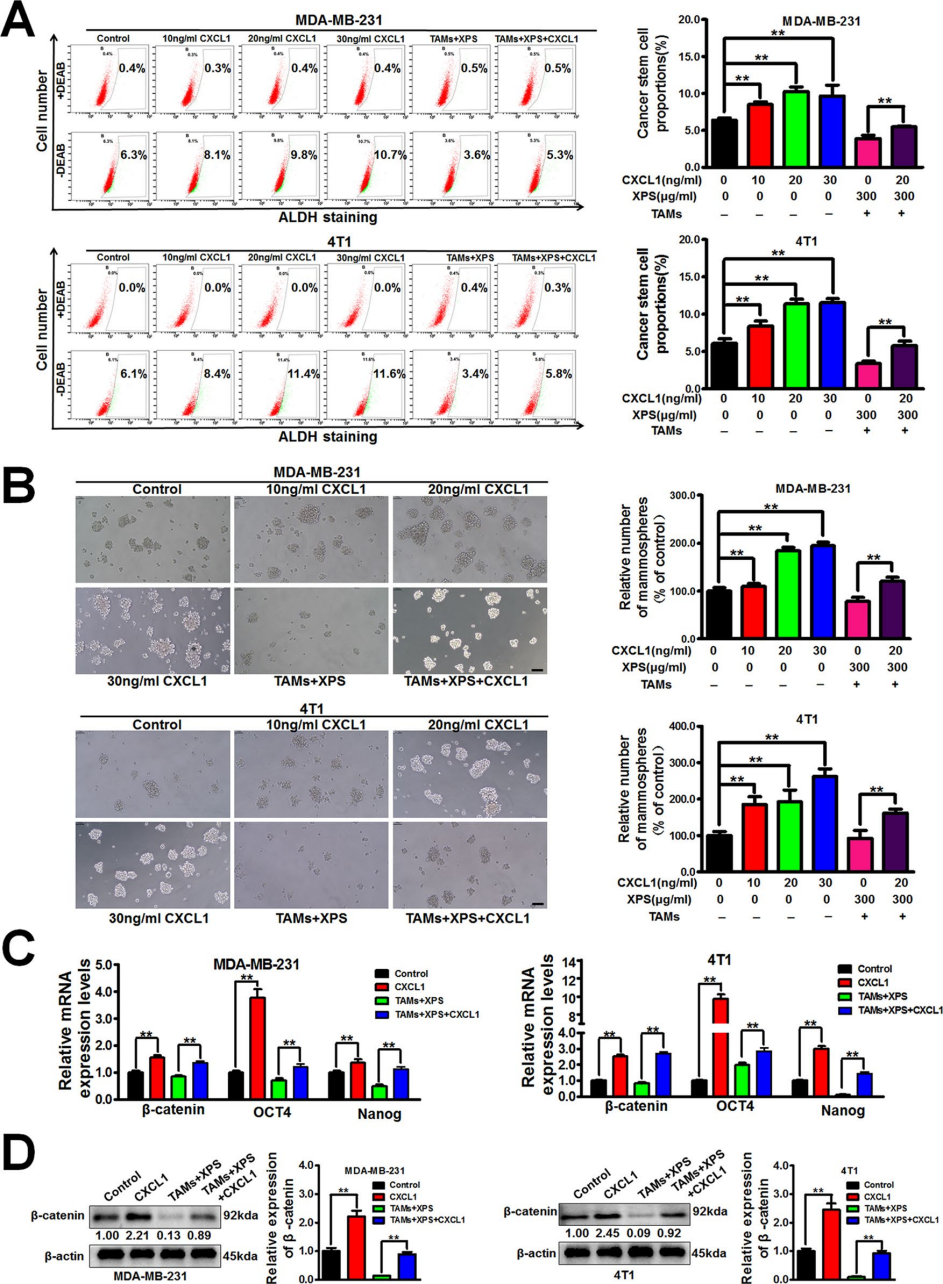
XIAOPI formula (XPS) suppresses breast cancer stem cells (CSCs) by modulating tumor-associated macrophages (TAMs)/C-X-C motif chemokine ligand 1 (CXCL1) pathway. **(A**-**B)** CXCL1 stimulation for 48 h could abrogate the inhibitory effect of XIAOPI formula on breast CSCs subpopulations and mammospheres formation abilities in the co-cultured breast cancer cells. Scale bar = 100 μm. **(C)** QPCR assay suggested that CXCL1 stimulation for 48 h could abrogate the inhibitory effect of XIAOPI formula on mRNA expression levels of stemness-related genes including *β-catenin*, *OCT4*, and *Nanog* in the co-cultured breast cancer cells. **(D)** Western blot assay further convinced that CXCL1 treatment for 48 h could reverse the inhibitory effect of XIAOPI formula on β-catenin expression in the co-cultured breast cancer cells. All values are presented as the mean ± SD, n = 3, **P < 0.05.

Considering that XPS could significantly inhibit CXCL1 expression and secretion from TAMs, it is important to further study whether CXCL1 is the key molecule involved in the inhibitory effect of XPS on breast CSCs. As shown in **Figures 4A, B**, CXCL1 administration could significantly increase the ALDH^+^ subpopulations and mammospheres numbers in breast cancer cells. Meanwhile, CXCL1 administration could abrogate the inhibitory effect of XPS on breast CSCs subpopulations and mammospheres formation abilities in the co-cultured breast cancer cells (**Figures 2A, B** and **Figures 4A, B**). QPCR assay further suggested that CXCL1 administration could elevate the mRNA expression levels of stemness-related genes, including *β-catenin, OCT4*, and *Nanog* in breast cancer cells while abrogating the inhibitory effect of XPS on mRNA expression levels of the above genes in the co-cultured breast cancer cells (**Figure 4C**). Western blot assay further convinced that CXCL1 administration could elevate β-catenin protein expression levels in breast cancer cells while reversing the inhibitory effect of XPS on β-catenin protein expression in the co-cultured breast cancer cells (**Figure 4D**). Altogether, XPS could inhibit the self-renewal activity of breast CSCs in the breast cancer cells and TAMs co-culture system by inhibiting TAMs/CXCL1 pathway.

### XPS Suppresses Breast Tumor Growth and Breast CSCs Activity *In Vivo*

**Figure 5:**
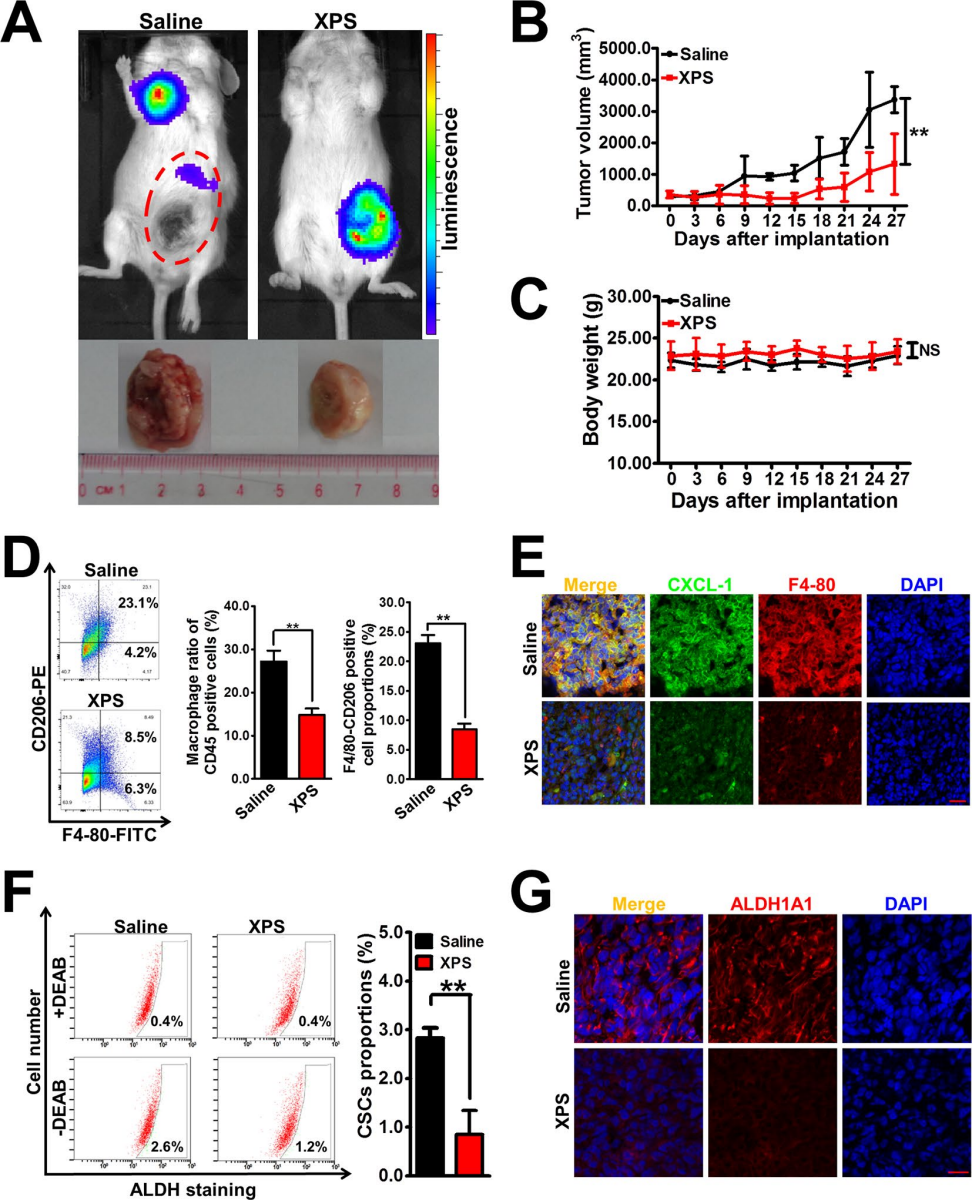
XIAOPI formula (XPS) suppresses breast tumor growth and breast cancer stem cells (CSCs) activity *in vivo*. **(A)** Representative pictures of the *in vivo* imaging experiments of mouse 4T1-Luc xenograft model. Breast cancer xenografts were established by implanting luciferase-labeled 4T1-Luc cells into the mammary glands of Balb/c mice. Mice bearing 4T1-Luc xenografts received either saline or XPS (1 g/kg/day) by intragastric perfusion. **(B)** XPS administration could effectively suppress mammary tumor growth in the mouse 4T1-Luc xenograft model *in vivo*. n = 6. **(C)** There were no significant differences in mouse body weights between the saline group and XIAOPI formula group, implying no additional toxic and side effects of XPS. n = 6. **(D–E)** XPS administration significantly decreased the infiltration degree of macrophages as well as their M2 phenotype polarization and C-X-C motif chemokine ligand 1 (CXCL1) expression in the 4T1-Luc xenografts *in vivo*. n = 3. (**F–G**) XPS administration significantly decreased the proportions of ALDH^+^ subpopulations (F) and suppressed the expression levels of ALDH1A1 **(G)** in the 4T1-Luc xenografts *in vivo*. n = 3. Scale bar = 20 μm. All values are presented as the mean ± SD, **P < 0.05.

Based on our *in vitro* findings, it is important to validate the anti-breast cancer activities and mechanisms of XPS *in vivo*. Breast cancer xenografts were established by implanting luciferase-labeled 4T1-Luc cells into the mammary glands of Balb/c mice. It was found that XPS administration could effectively suppress mammary tumor growth in the mouse 4T1-Luc xenograft model *in vivo* (**Figures 5A, B**). In addition, compared with the saline group, no treatment-correlated mortality or apparent decrease in body weight (**Figure 5C**) was observed, implying that XPS administration didn’t induce additional toxic and side effects. Notably, an *in vivo* imaging experiment also suggested that XPS could inhibit the metastasis efficacy of the breast cancer cell line 4T1-Luc (**Figure 5A**), suggesting XPS may also have anti-metastatic activity *in vivo*. Furthermore, XPS administration significantly decreased the infiltration degree of macrophages as well as their M2 phenotype polarization in the 4T1-Luc xenografts *in vivo*. Meanwhile, consistent with our *in vitro* findings, XPS administration strongly attenuated the expression of CXCL1 in mammary tumor tissue *in vivo* (**Figures 5D, E**). These results indicated that XPS might also inhibit M2 phenotype transformation and CXCL1 secretion of TAMs *in vivo*. Moreover, XPS administration significantly decreased the proportions of ALDH^+^ subpopulations and suppressed the expression of ALDH1A1 in the 4T1-Luc xenografts, indicating that XPS could also inhibit the activity of breast CSCs *in vivo* (**Figures 5F, G**). As shown in **Supplementary Figure 1** and **Supplementary Materials and Methods**, TAMs co-injection significantly increased mammary tumor growth as well as breast CSCs population when compared with saline group. Furthermore, both XPS treatment and CXCL1 knockdown in TAMs could partly abrogate the promotion effect of TAMs on mammary tumor growth and breast CSCs population. More importantly, CXCL1 overexpression in TAMs partly reversed the inhibitory effect of XPS on mammary tumor growth and breast CSCs population. Taken together, XPS could suppress breast tumor growth and breast CSCs activity *in vivo* by modulating TAMs/CXCL1 pathway.

## Discussion

Breast cancer is a serious threat to women’s health with high morbidity and mortality (Bray et al., 2018). For many decades, extensive efforts have been made to identify and develop novel targets or treatment strategies for breast cancer. Although a number of cytotoxic drugs or cytostatic drugs have been developed for breast cancer treatment, such as anthraquinones, alkylating agents, alkaloids, and antimetabolites, their therapeutic effects are disappointing due to the inter- and intra-tumor heterogeneity (Jin and Ye, 2013; Yuan et al., 2016). Lately, the traditional strategy has given way to the understanding of the tumor microenvironment, thus enabling modification of the immunological dynamics between tumor cells and their host (Kjeldsen et al., 2018). An era of new drugs aiming to unlock the host immune system is steadily coming. Emerging immunotherapy strategies including immune checkpoint inhibitors, therapeutic vaccines and adoptive cell therapy have brought incredible clinical efficacy and prolonged survival in “hard-to-treat” cancer types and previously refractory cases. In the present study, we proved that XPS, an existing anti-breast hyperplasia drug, could significantly inhibit breast CSCs *in vitro* by modulating M2 phenotype polarization and its CXCL1 secretion. What is more important, XPS could inhibit the growth of mouse 4T1-Luc xenografts *in vivo*. These results suggested that XPS could inhibit breast cancer *via* immunoregulation. This finding provided scientific evidence for the application of XPS in breast cancer treatment. In addition, this study also highlighted the tumor immune microenvironment as the critical mechanism contributing to the anti-cancer pharmacological activity of TCM.

CSCs are considered as the origin and driving force of cancer initiation and progression (Nilendu et al., 2017). The microenvironment of CSCs was consisted of a variety of components, such as immune cells. The complex interactions between immune cells and CSCs are being paid much attention nowadays. For instance, it was reported that CSCs exhibited lower immunogenicity when compared with non-CSCs, and they may inhibit multiple tumor-associated antigens, thus limiting the efficacy of the adaptive immune system to recognize them and result in cancer recurrence or metastasis (Di Tomaso et al., 2010; Chikamatsu et al., 2011; Volonte et al., 2014). CSCs could secrete a variety of molecules, such as TGF-β, which could promote Treg to iTreg differentiation and thus decrease its antitumor immunity (Dunn et al., 2004; Bruttel and Wischhusen, 2014; Maccalli et al., 2014). In addition, CSCs could recruit immunosuppressive immune cells to the tumor microenvironment and therefore favor their own survival (Vahidian et al., 2019). With regard to TAMs, it was reported that multiple factors including CSF-1 and CCL2 were enriched in the CSCs microenvironment, and could recruit macrophages polarized to the immune-suppressive M2 phenotype (Wu et al., 2010; Zhou et al., 2015). Additionally, TAMs could also mediate the function of CSCs in numerous ways (Jinushi et al., 2012; Yang et al., 2013). For example, it was reported that TAMs were necessary for preventing CSCs from the continuous immune surveillance in the tumor microenvironment (Wynn et al., 2013; Noy and Pollard, 2014; Sarkar et al., 2014). Furthermore, TAMs could activate the Hedgehog pathway in CSCs and promote proliferation and invasion of CSCs by releasing TGF-β and the stemness-favoring cytokines IL-8 and CXCL12 (Plaks et al., 2015). In the present study, we demonstrated that inhibiting CXCL1 secretion from TAMs is a novel tool for suppressing the self-renewal activity of breast CSCs. These results indicated that targeting CSCs by immunoregulation approaches may be a promising way to eliminate these tumor-initiating cells.

The immune cells in the tumor microenvironment, including TAMs, could release chemokines as inflammatory mediators influencing cancer progression by activating more immunosuppressive cells to the tumor stroma and finally promoting carcinogenesis and metastasis (De la Fuente Lopez et al., 2018). Chemokines are a large family of small cytokines and generally have low molecular weight ranging from 7 to 15 kDa with pleiotropic functions (cellular chemotaxis, cytokine secretion, cell proliferation, and survival). There are two major chemokine sub-families based upon cysteine residues position: CXC and CC. Nowadays, approximately 50 endogenous chemokines ligands have been described while CXCL1 is the most abundant chemokine secreted by TAMs (Palomino and Marti, 2015; Wang et al., 2018). Accumulating reports have indicated that CXCL1 is a promising marker for the detection and prognosis prediction of multiple cancers. For example, CXCL1 expression was upregulated in gastric cancer tissues when compared with adjacent noncancerous tissues. CXCL1 upregulation was one of the independent prognostic factors for gastric cancer patient’s survival and was significantly associated with tumor progression of gastric cancer patients (Cheng et al., 2011). In bladder cancer, urinary CXCL1 levels were significantly higher in patients with invasive bladder cancer than those with noninvasive tumors and normal controls, indicating CXCL1 was an independent factor for predicting bladder cancer invasion (Kawanishi et al., 2008). What is more important, high CXCL1 level in bladder cancer tissue was positively associated with increased TAMs infiltration and higher pathologic stages in bladder cancer (Kawanishi et al., 2008; Miyake et al., 2016). With regard to breast cancer, Zou et al. reported that increased CXCL1 expression in breast cancer stroma correlated with poor patient prognosis (Zou et al., 2014). We also have previously reported that CXCL1 derived from TAMs could promote the epithelial-mesenchymal transition, migration, and invasion of breast cancer cells *in vitro*. Additionally, CXCL1 silencing in TAMs could inhibit the growth and lung metastasis of breast cancer xenografts induced by TAMs *in vivo*. (Wang et al., 2018). Mechanism investigations furthered identified the NF-κB/SOX4 signaling as the major target of TAMs/CXCL1 in promoting breast cancer metastasis. This was in consistent with the existing report that CXCL1-mediated interaction between TAMs and cancer cells could promote tumor progression in human bladder cancer (Miyake et al., 2016). Additionally, TAMs-secreted CXCL1 could recruit MDSCs into the tumor microenvironment, which secreted chemokines including S100A8/9 that enhance cancer cell survival, chemoresistance, metastasis, and pre-metastatic niche formation (Acharyya et al., 2012; Miyake et al., 2016). In the present study, we found that TAMS-secreted CXCL1 could promote breast CSCs self-renewal activity. This was consistent with the existing report that inflammatory chemokine CXCL1 could enhance colon CSC characteristics by enhancing CD133 expression and aldehyde dehydrogenase activity (Hsu et al., 2018). Considering that breast CSCs are the origin cell of breast cancer initiation as well as the driving force of breast cancer progression, TAMs/CXCL1 pathway in the tumor microenvironment may also play a critical role in modulating breast cancer progression and prognosis and has potential to be developed as a diagnostic and prognostic biomarker for breast cancer in the future.

## Conclusion

In conclusion, XPS could inhibit breast cancer growth *via* interrupting CXCL1-mediated interaction between TAMs and CSCs. This study not only uncovers the immunomodulatory mechanism of XPS in treating breast cancer but also provides novel insights into TAMs/CXCL1 as a potential molecular target for breast CSCs elimination.

## Data Availability Statement

All datasets generated for this study are included in the article/**Supplementary Material**.

## Ethics Statement

The animal study was reviewed and approved by the Institutional Animal Care and Use Committee of Guangdong Provincial Hospital of Chinese Medicine.

## Author Contributions

ZW conceived and designed the experiments. SW, RH, YZ, NW, and BY carried out the experiments and wrote the manuscript. XL, HS, and YL took part in the discussion and proofreading the manuscript. All authors have reviewed and approved the final version of the manuscript.

## Funding

This work was supported by the National Natural Science Foundation of China (81573651, 81873306, 81973526, 81703749, 81703764), Guangdong Science and Technology Department (2016A030306025), Guangdong High-level Personnel of Special Support Program (A1-3002-16-111-003), Department of Education of Guangdong Province (2018KZDXM022 and A1-2606-19-111-009), Guangdong traditional Chinese medicine bureau project (20181132 and 20182044), the PhD Start-up Fund of Natural Science Foundation of Guangdong Province (2017A030310213 and 2018A030310506), Science and Technology Planning Project of Guangdong Province (2017B030314166), Guangzhou science and technology project (201904010407), the Specific Research Fund for TCM Science and Technology of Guangdong provincial Hospital of Chinese Medicine (YN2018MJ07, YN2018QJ08).

## Conflict of Interest

The authors declare that the research was conducted in the absence of any commercial or financial relationships that could be construed as a potential conflict of interest.

## Abbreviations

XPS, XIAOPI formula; TAMs, Tumor-associated macrophages; CSCs, Cancer stem cells; CXCL1, C-X-C motif chemokine ligand 1; TCM, Traditional Chinese Medicine; MDSCs, Myeloid-derived suppressor cells; Tregs, Regulatory T cells; CTLs, Cytotoxic T-lymphocytes; Ths, T helper cells; EGF, Epidermal growth factor; PMA, Phorbol-12-myristate-13-acetate; CM, Conditioned medium; DEAB, Diethylaminobenzaldehyde; BSA, Bovine serum albumin; CST, Cell Signaling Technology; DAPI, 4’, 6-diamidino-2-phenylindole; ALDH, Aldehyde dehydrogenase; OCT4, Octamer-binding transcription factor 4.
