# Supplementary material for: XIAOPI Formula Inhibits Breast Cancer Stem Cells via Suppressing Tumor-Associated Macrophages/C-X-C Motif Chemokine Ligand 1 Pathway
Source: Front Pharmacol. 2019 Nov 15;10:1371. doi: 10.3389/fphar.2019.01371 (PMC6874098; doi:10.3389/fphar.2019.01371)
Supplement: Supplementary file 1 [file DataSheet_1.pdf]

Supplementary Material

1 Supplementary Materials and Methods

All *in vivo* experiments were performed according to our institution's guidelines for the use of laboratory animals and were approved by the Institutional Animal Care and Use Committee of Guangdong Provincial Hospital of Chinese Medicine (No.2018044). Female Balb/c mice were raised at the Experimental Animal Center of Guangdong Provincial Hospital of Chinese medicine under specific pathogen-free conditions with ambient temperature of 20-25℃ and 45-50% relative humidity and given sterilized food and water. The animal assay was carried out to directly prove the role of TAMs-secreted CXCL1 on breast CSCs as well as the inhibitory effect of XIAOPI formula on breast CSCs by modulating TAMs/CXCL1 pathway. Briefly, the female Balb/c mice were randomized into 5 groups (n = 6 in each group) including saline group (2×10^6 4T1-Luc cells were inoculated subcutaneously into the mammary fat pads followed by saline treatment), TAMs group (co-injection of 2×10^6 4T1-Luc cells with 6×10^6 Raw264.7-derived TAMs followed by saline treatment), TAMs + XIAOPI formula group (2×10^6 4T1-Luc and 6×10^6 Raw264.7-derived TAMs co-injection followed by XIAOPI formula treatment), TAMs/shCXCL1 group (co-injection of 2×10^6 4T1-Luc and 6×10^6 Raw264.7-derived TAMs with CXCL1 knockdown, followed by saline treatment), TAMs/rCXCL1 + XIAOPI formula group (co-injection of 2×10^6 4T1-Luc and 6×10^6 Raw264.7-derived TAMs with CXCL1 overexpression, followed by XIAOPI formula treatment). When tumors reached a mean diameter of 0.5 cm, saline or XIAOPI formula treatment (1 g/kg/day) were administrated by intragastric perfusion as indicated above. When tumors grew to a proper size, mice were euthanized and tumors were excised and weighed. Primary breast cancer cells were isolated from fresh mammary tumors by mechanical methods and subjected for breast CSCs subpopulation analysis using the ALDEFLUOR Stem Cell Identification Kit (01700, STEMCELL Technologies, Vancouver, Canada) according to the manufacturer's instructions.

2 Supplementary Figures

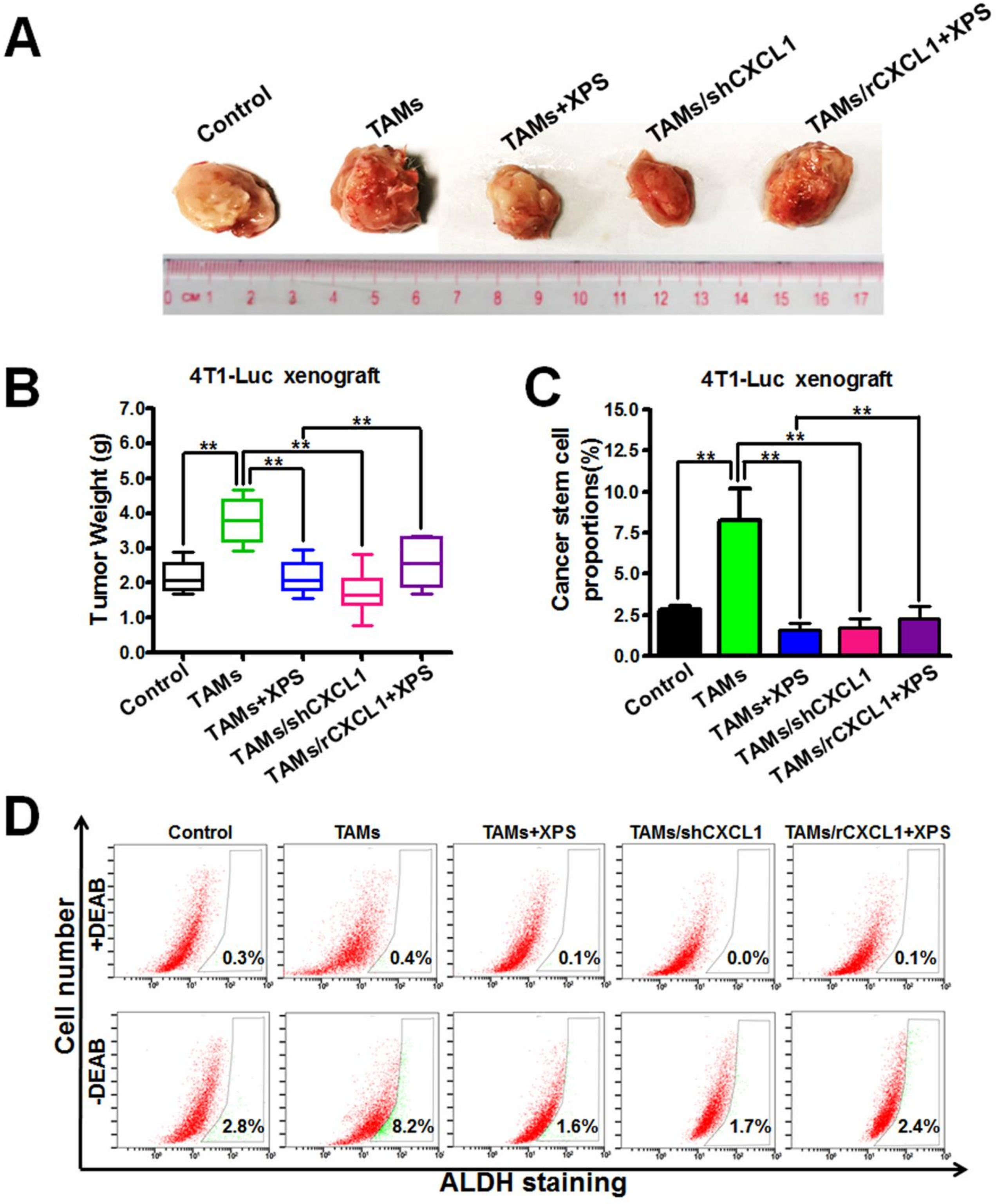

Supplementary Figure 1. The weights of tumors as well as the proportions of $ALDH^+$ subpopulations in different groups. (**A**) The representative pictures of mouse mammary tumors in different groups. (**B**) The statistical analysis of tumor weights in different groups. (**C-D**) The proportions of $ALDH^+$ subpopulations in total primary breast cancer cells in different groups. Primary breast cancer cells were isolated from fresh mammary tumors and subjected for breast CSCs subpopulation analysis by the $ALDH^+$ staining assay. All values are presented as the mean ± SD, n = 6, $^{**}P < 0.05$.
